# High interindividual variability in LDL-cholesterol reductions after inclisiran administration in a real-world multicenter setting in Germany

**DOI:** 10.1007/s00392-023-02247-8

**Published:** 2023-07-09

**Authors:** U. Makhmudova, U. Schatz, N. Perakakis, U. Kassner, F. Schumann, C. Axthelm, P. Stürzebecher, D. L. Sinning, A. Doevelaar, B. Rohn, T. Westhoff, A. Vogt, M. Scholl, U. Kästner, J.-A. Geiling, K. Stach, J. Mensch, E. Lorenz, C. Paitazoglou, I. Eitel, A. Baessler, E. Steinhagen-Thiessen, W. Koenig, P. C. Schulze, U. Landmesser, U. Laufs, Oliver Weingärtner, for the German Inclisiran Network (GIN), U. Makhmudova, U. Schatz, N. Perakakis, U. Kassner, F. Schumann, C. Axthelm, P. Stürzebecher, D. L. Sinning, A. Doevelaar, B. Rohn, T. Westhoff, A. Vogt, M. Scholl, U. Kästner, J.-A. Geiling, K. Stach, J. Mensch, E. Lorenz, C. Paitazoglou, I. Eitel, A. Baessler, E. Steinhagen-Thiessen, W. Koenig, P. C. Schulze, U. Landmesser, U. Laufs, Oliver Weingärtner

**Affiliations:** 1 Department of Internal Medicine I, Division of Cardiology, Angiology and Intensive Medical Care, Friedrich-Schiller-University, University Hospital Jena, Am Klinikum 1, 07747 Jena, Germany; 2 Department of Internal Medicine III, University Hospital Carl Gustav Carus, Technical University of Dresden, Dresden, Germany; 3 Paul Langerhans Institute Dresden (PLID), Helmholtz Center Munich, University Hospital and Faculty of Medicine, TU Dresden, Dresden, Germany; 4 German Center for Diabetes Research (DZD E.V.), Neuherberg, Germany; 5 Clinic for Endocrinology and Metabolic Medicine, Charité-University Medicine Berlin, Berlin, Germany; 6 Cardiologicum Dresden and Pirna, Dresden, Germany; 7 Department of Cardiology, University Hospital Leipzig, Leipzig, Germany; 8 Deutsches Herzzentrum der Charité, Department of Cardiology, Angiology and Intensive Care Medicine, Berlin, Germany; 9 Friede Springer Cardiovascular Prevention Center at Charité, Charité Universitätsmedizin Berlin, Berlin, Germany; 10 Medical Clinic I, Marien Hospital Herne, University Hospital of the Ruhr-University of Bochum, Herne, Germany; 11 Department of Internal Medicine IV, University Hospital Munich, Munich, Germany; 12 Medical Care Centre, Nephrocare Mühlhausen GmbH, Mühlhausen/Thuringia, Germany; 13 Department of Internal Medicine V, University Hospital Mannheim, Mannheim, Germany; 14 Institute for Clinical Chemistry, University Medicine Rostock, Rostock, Germany; 15 Deutsches Herzzentrum München, Technical University Munich, Munich, Germany; 16 Department of Internal Medicine II, University Hospital Schleswig-Holstein, Lübeck, Germany; 17 German Centre for Cardiovascular Research (DZHK), Partner Site Hamburg-Kiel-Lübeck, Lübeck, Germany; 18 Department of Internal Medicine II, University Hospital Regensburg, Regensburg, Germany; 19 German Centre for Cardiovascular Research (DZHK), Partner Site Munich Heart Alliance, Munich, Germany; 20 Institute of Epidemiology and Medical Biometry, University of Ulm, Ulm, Germany; 21 Charité—Universitätsmedizin Berlin, Corporate member of Freie Universität Berlin and Humboldt-Universität zu Berlin, Berlin, Germany, Berlin, Germany

**Keywords:** Low-density lipoprotein cholesterol, Inclisiran, PCSK9, siRNA

## Abstract

**Background and aims:**

Low-density lipoprotein cholesterol (LDL-C) is the main therapeutic target in the treatment of hypercholesterolemia. Small interfering RNA (siRNA) inclisiran is a new drug, which targets PCSK9 mRNA in the liver, reducing concentrations of circulating LDL-C. In randomized trials, inclisiran demonstrated a substantial reduction in LDL-C. The German Inclisiran Network (GIN) aims to evaluate LDL-C reductions in a real-world cohort of patients treated with inclisiran in Germany.

**Methods:**

Patients who received inclisiran in 14 lipid clinics in Germany for elevated LDL-C levels between February 2021 and July 2022 were included in this analysis. We described baseline characteristics, individual LDL-C changes (%) and side effects in 153 patients 3 months (n = 153) and 9 months (n = 79) after inclisiran administration.

**Results:**

Since all patients were referred to specialized lipid clinics, only one-third were on statin therapy due to statin intolerance. The median LDL-C reduction was 35.5% at 3 months and 26.5% at 9 months. In patients previously treated with PCSK9 antibody (PCSK9-mAb), LDL-C reductions were less effective than in PCSK9-mAb-naïve patients (23.6% vs. 41.1% at 3 months). Concomitant statin treatment was associated with more effective LDL-C lowering. There was a high interindividual variability in LDL-C changes from baseline. Altogether, inclisiran was well-tolerated, and side effects were rare (5.9%).

**Conclusion:**

In this real-world patient population referred to German lipid clinics for elevated LDL-C levels, inclisiran demonstrated a high interindividual variability in LDL-C reductions. Further research is warranted to elucidate reasons for the interindividual variability in drug efficacy.

**Graphical abstract:**

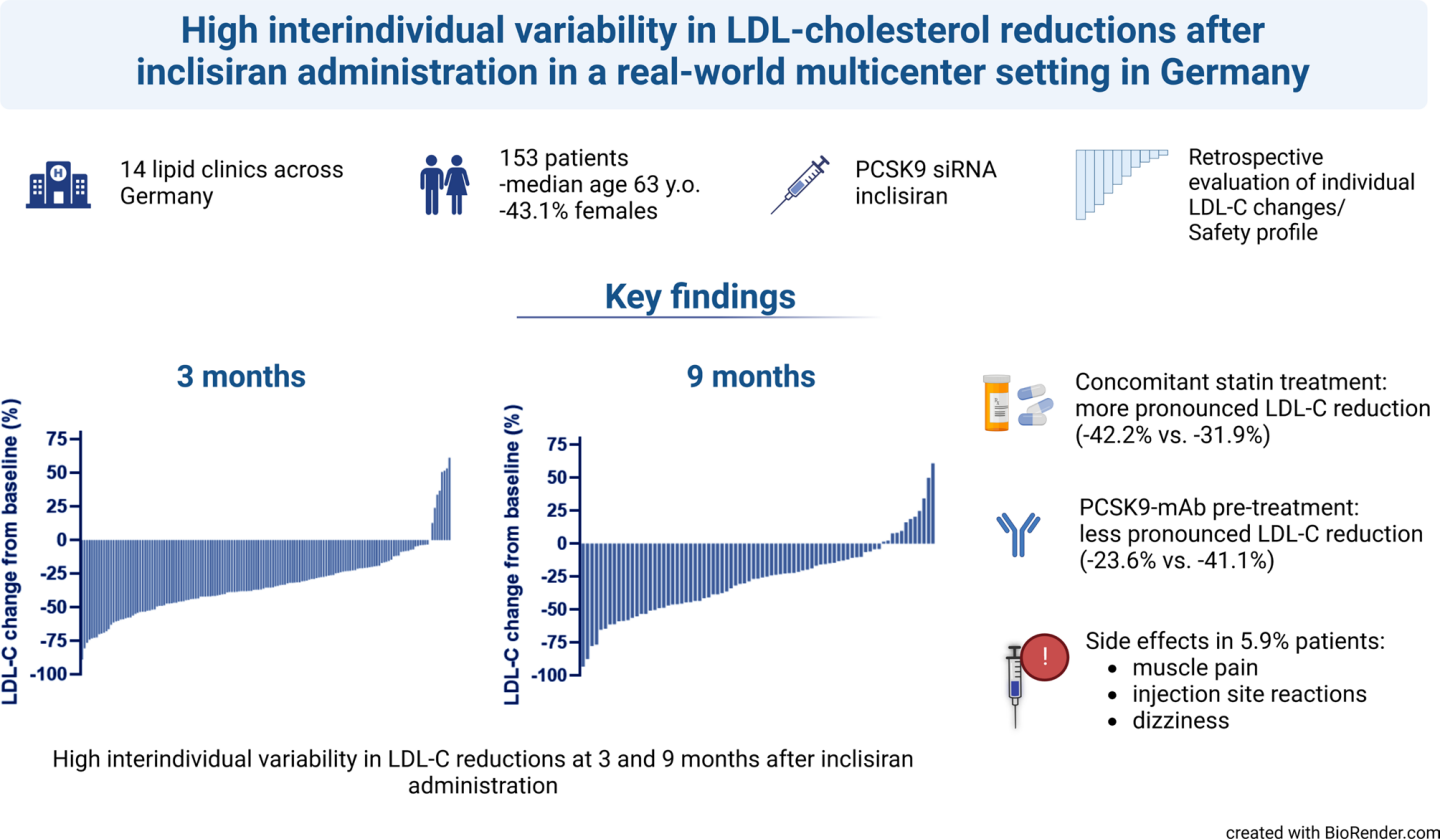

**Supplementary Information:**

The online version contains supplementary material available at 10.1007/s00392-023-02247-8.

## Introduction

Elevated low-density lipoprotein (LDL-C) cholesterol concentrations are a causal risk factor for atherosclerotic cardiovascular disease (ASCVD). The European Society of Cardiology (ESC) and the European Atherosclerosis Society (EAS) released updated guidelines for the management of elevated cholesterol levels in 2020 [1]. Statins are first-line lipid-lowering therapy (LLT) for patients with elevated cholesterol levels. When LDL-C targets cannot be achieved, lipid-lowering therapy should be escalated accordingly with either ezetimibe or proprotein convertase subtilisin/kexin type 9 inhibitors (PCSK9-mAb).

The inhibition of PCSK9 messenger RNA (mRNA) is an emerging lipid-lowering concept [2–4. PCSK9 is produced in the liver and binds to LDL-C receptors at the surface of hepatocytes, which leads to the inhibition of LDL receptor (LDL-R) recycling and enhanced degradation [5]. Inclisiran is a first-in-class small interfering RNA (siRNA) conjugated to triantennary N-acetylgalactosamine carbohydrates (GalNAc) which targets PCSK9 mRNA [6].

The first approval for the siRNA inclisiran was given by the European Medicine Agency in December 2020 [6] for the treatment of adults with hypercholesterolemia or mixed dyslipidemia. The Food and Drug Administration approved inclisiran in 2021. In Germany, inclisiran has been available since February 2021 and can be prescribed by cardiologists, nephrologists, endocrinologists, angiologists, and doctors working in lipid clinics for patients with hypercholesterolemia or mixed dyslipidemia.

Against the background of reported high interindividual variabilities in LDL-C reductions with statins, ezetimibe, PCSK9-mAb and bempedoic acid [7–10, we hypothesized that cholesterol lowering with inclisiran exhibits a similar substantial interindividual variability in lowering LDL-C. Therefore, the aim of this retrospective, multicenter analysis was to use individual patient data to determine the extent of the variabilities in LDL-C reductions in response to inclisiran administration in patients treated with inclisiran in Germany.

## Methods

The German Inclisiran Network (NCT05438069) includes 14 lipid clinics in Germany (Supplementary Table 1). Electronic data records of patients treated with inclisiran (Supplementary Fig. 1, Supplementary Table 1) were collected from February 2021 to July 2022. In contrast to patients included in the ORION study program, inclisiran was administered to a broader range of patients with elevated LDL-C, including patients with statin intolerance as well as patients on statins, ezetimibe, bempedoic acid and on lipoprotein apheresis. The study was approved by the Local Ethic Committee of the Jena University Hospital (2021-2429).

Patients with changes in lipid-lowering medications or administration of PCSK9-mAbs within 4 weeks prior to inclisiran administration were excluded from the analysis. Reasons for PCSK9 discontinuation, such as poor response and poor tolerance, were defined individually by lipid specialists. The term “poor response” refers to a situation with an inadequate reduction in LDL-C levels following the administration of PCSK9-mAb. Similarly, poor PCSK9-mAb tolerability refers to the occurrence of side effects significant enough to cause discontinuation of PCSK9-mAbs. We also excluded patients who changed background LLT after inclisiran administration. Overall, the study included 153 patients. All patients were followed-up at 3 months after the first inclisiran administration. Of them, a total of 79 patients were followed-up both at 3 and 9 months after the first administration of inclisiran (Supplementary Fig. 1). Inclisiran was injected in the respective lipid clinics by qualified medical professionals in accordance with the Medicinal Products Directives established by the German Federal Joint Committee [11].

The median LDL-C response was calculated as percentage change from baseline. All statistical analyses were conducted with R Statistics (Version 4.1.2), and statistical significance was assessed at a 2-sided 5% level. Statistical significances were calculated using the Wilcoxon rank sum test (for non-normal distribution) and Student’s t-test (for normal distribution) to compare differences between two groups of continuous variables. The Kruskal–Wallis test was used to compare more than two groups. The normality of distribution was tested using histograms and Shapiro–Wilk test.

Spearman correlation coefficient was used to determine the correlation between LDL-C change from baseline and other variables. Multiple regression model included LDL-C change from baseline (%) as dependent variable and sex, age, baseline LDL-C, ASCVD, PCSK9-mAb treatment and concomitant treatment with statins/ezetimibe as independent variables. For the final model, the R-squared was 0.15, F-statistic 4.24 on six and 146 degrees of freedom. Graphs were created using GraphPad Prism 9.5.0 and R Statistics. Supplementary Fig. 1 was created with BioRender.com.

## Results

### Patient characteristics

Patients were on average 63.0 (IQR 55.0; 70.0) years old, and 66 (43.1%) were female. Median LDL-C concentration at baseline was 3.6 mmol/L (IQR 2.4; 4.8), or 139.2 mg/dL (IQR 92.8; 185.6), respectively *(*Table 1*)*.

**Table 1:** Baseline characteristics of patients

Variable	Total N = 153	PCSK9-mAb n = 58	No PCSK9-mAb n = 95
Age, median [IQR]	63.0 (55.0; 70.0)	64.0 (57.0; 71.8)	63.0 (55.0; 68.5)
Females, n (%)	66 (43.1)	30 (51.7)	36 (37.9)
Males, n (%)	87 (56.9)	28 (48.3)	59 (62.1)
Baseline LDL-C			
In mmol/L, median (IQR)	3.6 (2.4; 4.8)	4.0 (2.8; 5.2)	3.4 (2.3; 4.4)
In mg/dL, median (IQR)	139.2 (92.8; 185.6)	154.7 (108.3; 201.1)	131.5 (88.9; 170.2)
Baseline TC			
In mmol/L, median (IQR)	5.7 (4.4; 6.8)	6.1 (4.6; 7.2)	5.2 (4.2; 6.6)
In mg/dL, median (IQR)	220.4 (170.1; 263.0)	235.9 (177.9; 278.4)	201.1 (162.4; 255.2)
Baseline HDL-C			
In mmol/L, median (IQR)	1.3 (1.1; 1.6)	1.4 (1.0; 1.6)	1.3 (1.1; 1.5)
In mg/dL, median (IQR)	50,3 (42.5; 61.9)	54.1 (38.7; 61.9)	50.3 (42.5; 58.0)
Baseline TG			
In mmol/L, median (IQR)	1.7 (1.2; 2.9)	1.9 (1.3; 3.1)	1.7 (1.2; 2.7)
In mg/dL, median (IQR)	150.6 (106.3; 256.9)	168.3 (115.1; 274.6)	150.6 (106.3; 239.1)
ASCVD, n (%)	128 (83.6)	50 (86.2)	78 (82.1)
diabetes mellitus, n (%)	30 (19.7)	14 (24.1)	16 (16.8)
FH, n (%)	72 (47.1)	23 (39.7)	49 (52.1)
Chronic kidney disease^a^, n (%)	26 (17.0)	2 (3.4)	24 (25.3)
Thyroid disease, n (%)	32 (20.9)	7 (12.1)	25 (26.3)
Hypothyreodism	28 (18.3)	6 (10.3)	22 (23.2)
Hyperthyreodism	4 (1.3)	1 (1.7)	3 (3.2)
Liver steatosis, n (%)	26 (17.0)	2 (3.4)	24 (25.3)

*PCSK9-mAb* proprotein convertase subtilisin/kexin type 9 monoclonal antibody, *ASCVD* atherosclerotic cardiovascular disease, *FH* familial hypercholesterolemia, *TC* total cholesterol, *HDL-C* high-density lipoprotein cholesterol, *TG* triglycerides

^a^Defined as per KDIGO criteria for chronic kidney disease

We analyzed two cohorts separately: patients who had received PCSK9-mAb in the past (n = 58) and PCSK9-mAb naïve patients (n = 95). PCSK9-mAb pre-treatment was characterized by higher baseline LDL-C concentrations, more female patients (Table 1), and less background lipid-lowering therapy (Table 2). Fifty-eight patients (37.9%) had received treatment with PCSK9-mAb in the past and were switched to inclisiran due to PCSK9-mAb intolerance or poor LDL-C response (Table 2). Most patients (51/58) had a wash-out period of at least 3 months between PCSK9-mAb and inclisiran. Seven patients stopped PCSK9-mAb for at least 4 weeks prior to inclisiran administration. Of the 58 patients pre-treated with PCSK9-mAbs, 49/58 received evolocumab (140 mg or 420 mg) and 9/58 received alirocumab (75 mg or 150 mg). Eighty-three patients (54.2%) were on oral lipid-lowering therapy at baseline. Fifty-one patients (33.3%) received a combination of oral lipid-lowering drugs, while 32 (20.9%) received either statin, ezetimibe or bempedoic acid as monotherapy (Table 2). Twenty patients (13.1%) were on apheresis, either as monotherapy (7/20) or in combination with oral agents (13/20). The indication for apheresis were either elevated lipoprotein(a) levels (14/20) or high LDL-C levels not treatable with available drugs (6/20). Of note, 70 patients (45.8%) were not on any oral LLT at baseline due to statin intolerance and side effects of other lipid-lowering therapies. Familial hypercholesterolemia (FH) was diagnosed in approximately 47.1% of the patients, as per the Dutch Network Score criteria or confirmed by genetic testing.

**Table 2:** Lipid-lowering therapy at baseline

Variable	Total n = 153	PCSK9-mAb n = 58	No PCSK9-mAb n = 95
Background lipid-lowering therapy			
Yes	83 (54.2)	24 (41.4)	59 (62.1)
No	70 (45.8)	34 (58.6)	36 (37.9)
Statin (total)	48 (31.4)	10 (17.2)	38 (40.0)
High-intensity	36 (23.5)	7 (12.1)	29 (30.5)
Moderate-intensity	3 (2.0)	1 (1.7)	2 (2.1)
Low-intenstiy	9 (5.9)	2 (3.4)	7 (7.4)
Ezetimibe (total)	64 (41.8)	14 (24.1)	50 (52.6)
Bempedoic acid (total)	31 (20.3)	11 (19.0)	20 (21.1)
Statin only	7 (4.6)	2 (3.4)	5 (5.3)
Ezetimibe only	15 (9.8)	5 (8.6)	10 (10.5)
Bempedoic acid only	10 (6.5)	8 (13.8)	2 (2.1)
Statin + ezetimibe	30 (19.6)	6 (10.3)	24 (25.3)
Statin + bempedoic acid	2 (1.3)	0	2 (2.1)
Ezetimibe + bempedoic acid	10 (6.5)	1 (1.7)	9 (9.5)
Statin + ezetimibe + bempedoic acid	9 (5.9)	2 (3.4)	7 (7.4)
Apheresis	20 (13.1)	8 (13.8)	12 (12.6)
Alone	7 (4.6)	2 (3.4)	5 (5.3)
In combination with oral LLT	13 (8.5)	6 (10.3)	7 (7.4)

All values shown as n (%)

*PCSK9-mAb* proprotein convertase subtilisin/kexin type 9 monoclonal antibody, *LLT* lipid-lowering therapy

### LDL-C change from baseline

**Fig. 1:**
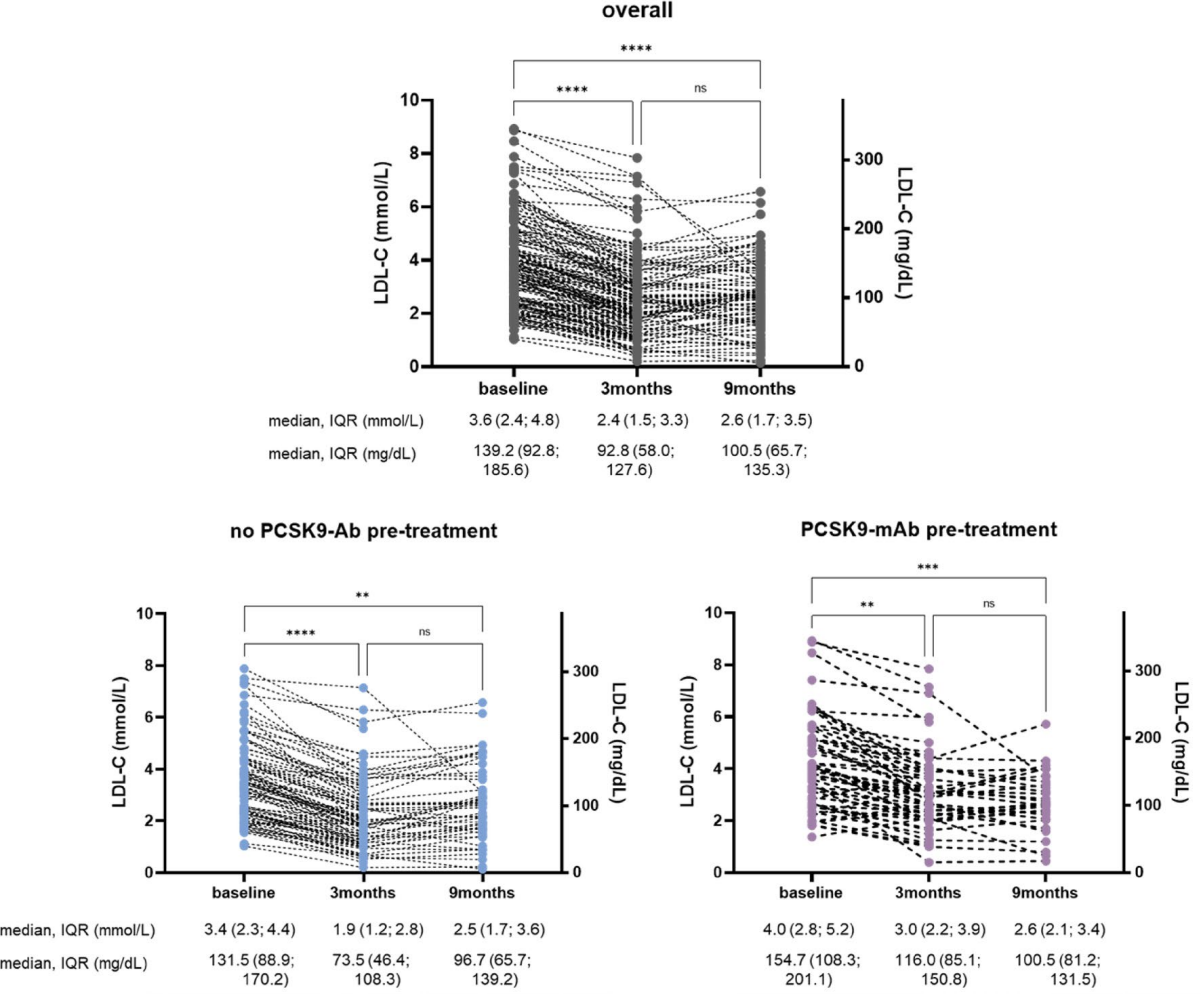
LDL concentration on baseline, 3 and 9 months after inclisiran administration shown as individual data points for the whole cohort (overall), PCSK9-mAb naïve, and PCSK9-mAb pre-treated patients. **p < 0.01, ***p < 0.001, ****p < 0.0001. *PCSK9-mAb* proprotein convertase subtilisin/kexin type 9 monoclonal antibody, *IQR* interquartile range

**Fig. 2:**
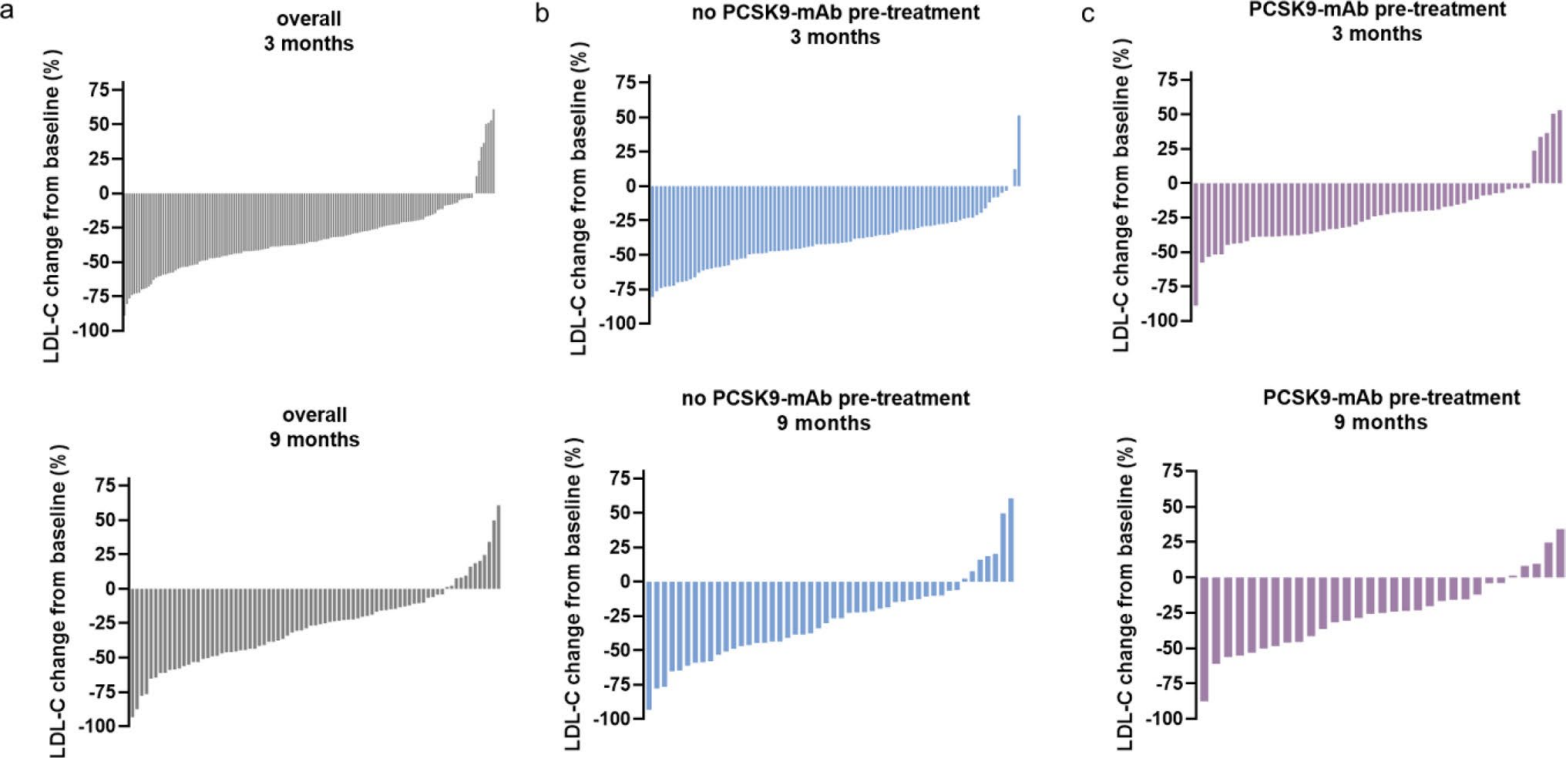
Waterfall plots depicting LDL-C change from baseline (%) in the overall cohort (overall) and in patients with or without PCSK9-mAb history at 3 and 9 months. LDL-C change from baseline was calculated as percent change from the baseline LDL-C value for each patient. *PCSK9-mAb* proprotein convertase subtilisin/kexin type 9 monoclonal antibody

**Fig. 3:**
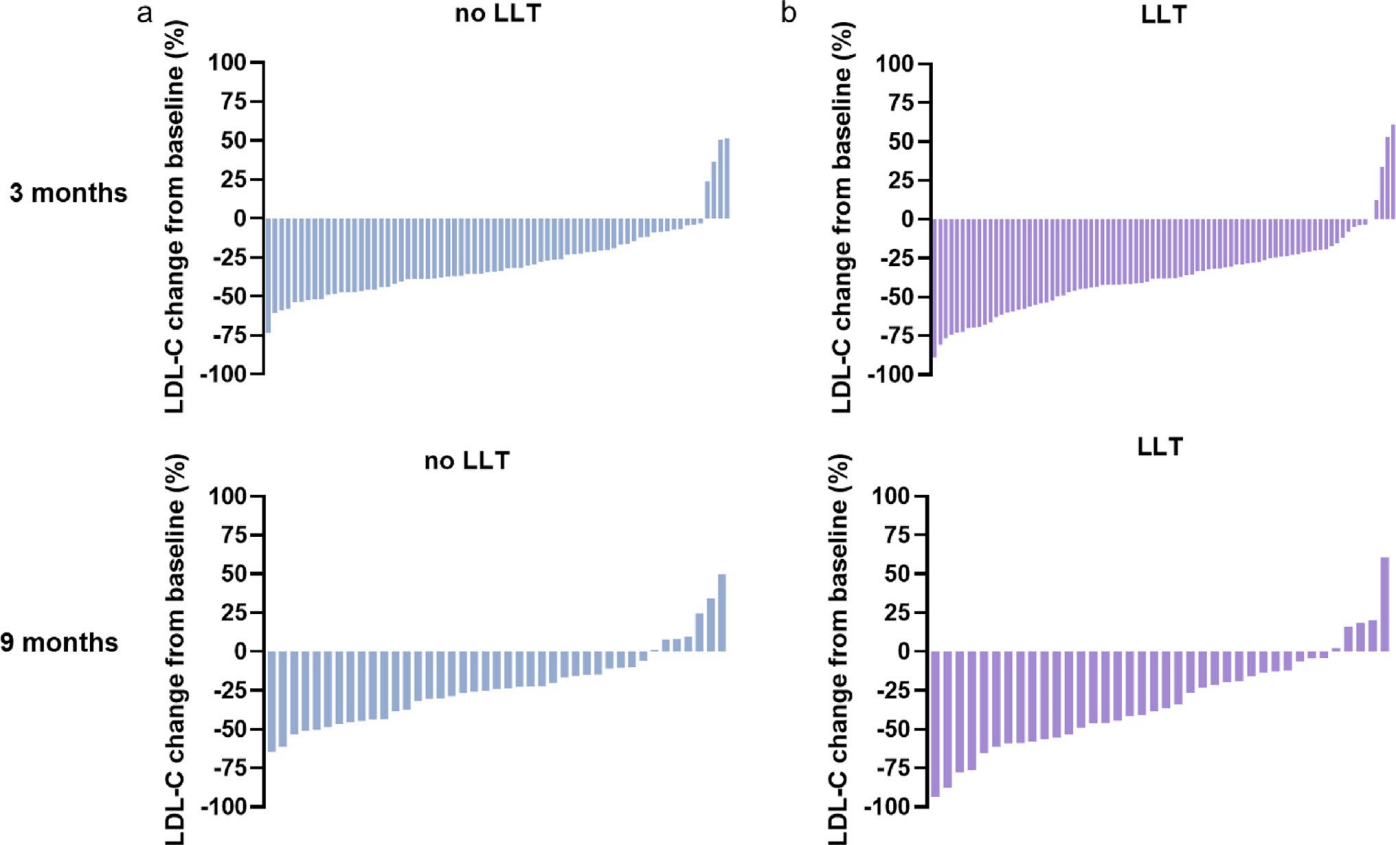
Waterfall plots depicting LDL-C change from baseline (%) in patients without concomitant LLT (**a**) and with concomitant LLT (**b**) at 3 and 9 months. *LLT* lipid-lowering therapy

In patients who did not receive PCSK9-mAb treatment prior to inclisiran, LDL-C was reduced from 3.4 mmol/L (131.5 mg/dL) at baseline to 1.9 mmol/L (73.5 mg/dL) at 3 months and 2.5 mmol/L (96.7 mg/dL) at 9 months (absolute change of 1.5 mmol/L (58 mg/dL) and 0.9 mmol/L (34.8 mg/dL), respectively), Fig. 1. Waterfall plots demonstrate a high interindividual variability in LDL-C reductions both at 3 and 9 months (Figs. 2, 3, Supplementary Fig. 2). The median individual LDL-C reduction was − 41.1% [95% confidence interval (CI), − 45.5; − 35.4] at 3 months (Table 3) and − 28.4% (95% CI, − 38.5; − 21.4) at 9 months.

**Table 3:** LDL-C change from baseline (%) at 3 months in different subgroups

Subgroups	Overall n = 153	PCSK9-mAb n = 58	No PCSK9-mAb n = 95
Overall	− 35.5 (− 38.7; − 31.6)	− 23.6 (− 33.3; − 20.0)	− 41.1 (− 45.5; − 35.4)
Sex			
Male	− 36.9 (− 42.1; − 32.3)	− 24.5 (− 33.3; − 19.1)	− 42.0 (− 47.1; − 35.4)
Female	− 35.6 (− 40.4; − 24.0)	− 23.6 (− 38.0; − 15.5)	− 40.6 (− 46.6; − 27.6)
Age			
< 65 years	− 36.2 (− 41.1; − 28.9)	− 22.9 (− 35.5; − 19.1)	− 41.6 (− 47.3; − 35.4)
≥ 65 years	− 35.4 (− 38.7; − 30.4)	− 27.2 (− 38.0; − 14.5)	− 37.4 (− 45.9; − 31.8)
LLT			
Yes	− 38.0 (− 42.1; − 31.8)	− 30.1 (− 38.0; − 19.7)	− 41.6 (− 48.9; − 35.4)
No	− 33.8 (− 37.8; − 26.4)	− 22.0 (− 35.5; − 12.1)	− 37.4 (− 46.6; − 31.9)
Overall statin			
Yes	− 42.2 (− 54.1; − 36.1)	− 40.0 (− 53.4; − 20.7)	− 43.1 (− 57.6; − 35.4)
No	− 31.9 (− 36.9; − 26.4)	− 21.2 (− 31.6; − 16.7)	− 37.9 (− 44.6; − 31.9)
FH			
Yes	− 34.7 (− 38.6; − 28.9)	− 24.0 (− 38.0; − 8.9)	− 37.8 (− 42.2; − 30.4)
No	− 37.0 (− 41.1; − 30.8)	− 23.3 (− 35.5; − 19.7)	− 43.8 (− 48.9; − 36.9)
Apheresis			
Yes	− 37.0 (− 51.2; − 16.7	− 10.5 (− 88.9; 53.3)	− 37.4 (− 67.8; − 24.8)
No	− 36.5 (− 40.4; − 31.6)	− 25.2 (− 35.5; − 20.2)	− 41.6 (− 45.6; − 35.4)

Data shown as median and 95% confidence interval (95% CI)

*PCSK9-mAb* proprotein convertase subtilisin/kexin type 9 monoclonal antibody, *LLT* lipid-lowering therapy, *FH* familial hypercholesterolemia

Baseline LDL-C in PCSK9-mAb pre-treated patients was 4.0 mmol/L (154.7 mg/dL) and LDL-C was reduced to 3.0 mmol/L (116.0 mg/dL) at 3 months and 2.6 mmol/L (100.5 mg/dL) at 9 months, Fig. 1. The median LDL-C change was − 23.6% (95% CI, − 33.3; − 20.0) at 3 months and − 25.1% (95% CI, − 41.4; − 15.7) at 9 months.

**Fig. 4:**
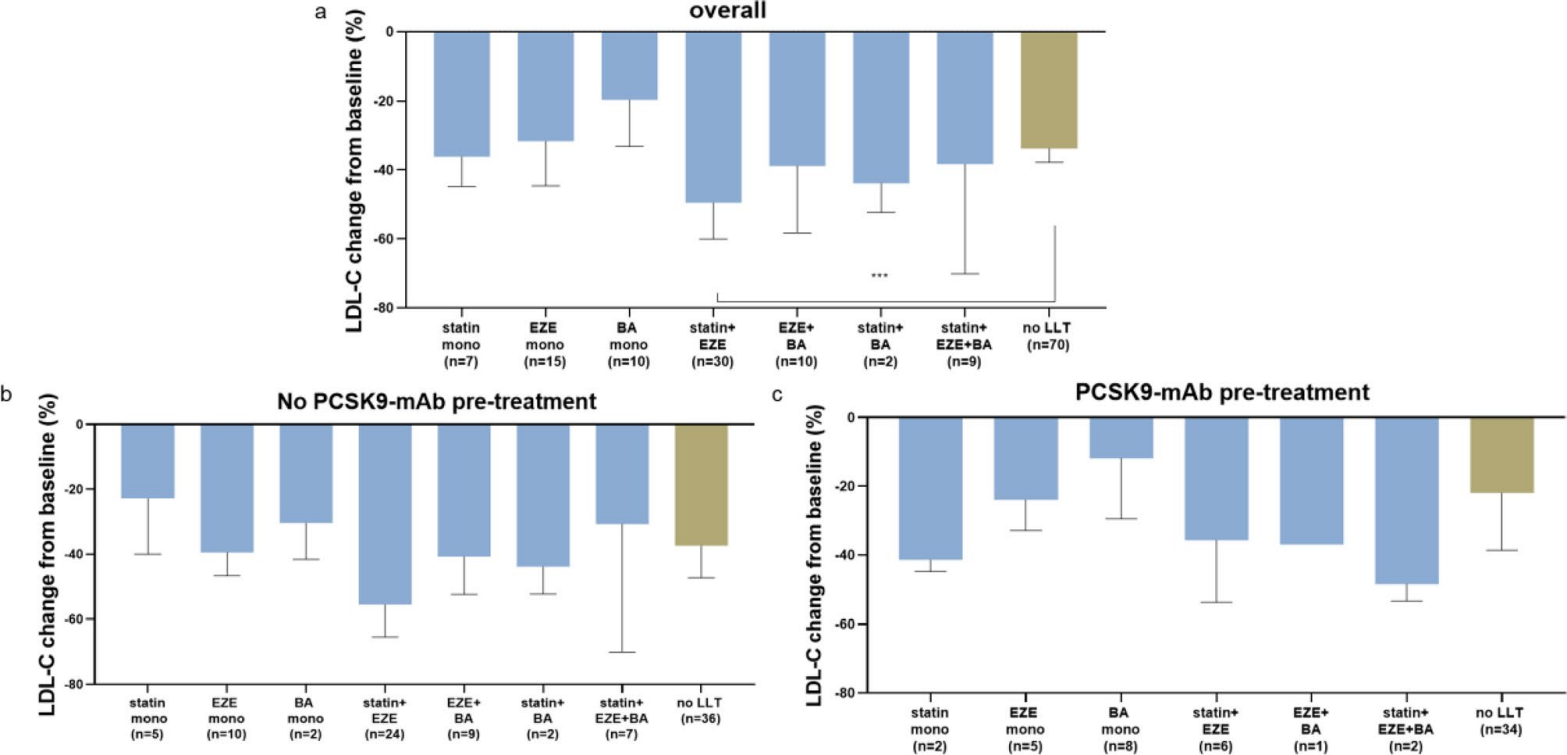
LDL change from baseline (%) at 3 months in different groups of concomitant LLT: overall (**a**), in patients not pre-treated with PCSK9-mAb (**b**) and in patients previously treated with PCSK9 mAb (**c**). Bars shown as median and IQR. ***p < 0.001. *PCSK9-mAb* proprotein convertase subtilisin/kexin type 9 monoclonal antibody, *BA* bempedoic acid, *EZE* ezetimibe, *LLT* lipid-lowering therapy

Further, we analyzed individual LDL-C reductions depending on background lipid-lowering therapy (LLT). This cohort included a variety of lipid-lowering strategies. Overall concomitant LLT and statin therapy were associated with more effective LDL-C reductions, especially in patients not pre-treated with PCSK9-mAb (Table 3). Of note, the use of combination therapies rather than monotherapies resulted in more pronounced LDL-C lowering (Fig. 4).

Spearman correlation coefficients between LDL-C change from baseline (%) and other variables showed that any oral LLT (r = − 0.16, p = 0.045) as well as statin or ezetimibe treatment (r = − 0.24, p = 0.003) were associated with more effective LDL-C reductions. Vice versa, baseline LDL-C (r = 0.17, p = 0.034) and PCSK9-mAb (r = 0.37, p = 0.000002) therapy were positively correlated with LDL-C changes (i.e., worse effectiveness) (Supplementary Fig. 4). There was no significant correlation with age or sex. In a multiple regression model, including sex, age, baseline LDL-C, ASCVD, concomitant statin or ezetimibe treatment and PCSK9-mAb pre-treatment, only statin or ezetimibe treatment (β = − 12.1; t = − 2.7; p = 0.0075) and PCSK9-mAb pre-treatment (β  = 13.1, t = 3.1, p = 0.002) were significant predictors of LDL-C change from baseline (%) 3 months after the first inclisiran injection (Table 4).

**Table 4:** Summary of multiple regression model predicting relative LDL-C change from baseline (%) at 3 months

Variable	β	Std. error	t-Value	p-Value
(Intercept)	− 28.9707	17.03763	− 1.7004	0.091185
Sex	− 1.54999	3.994785	− 0.388	0.698578
Age	0.068322	0.188089	0.363244	0.716948
Baseline LDL-C	− 1.58192	1.372067	− 1.15295	0.250817
PCSK9-mAb	13.0769	4.221508	3.097685	0.00234**
Statin or ezetimibe	− 12.099	4.465612	− 2.70937	0.007548**
ASCVD	1.372333	5.53218	0.248064	0.804434

Residual standard error: 23.61 on 146 degrees of freedom

Multiple R-squared: 0.1484, adjusted R-squared: 0.1134

F-statistic: 4.24 on 6 and 146 DF, p-value: 0.000577

*ASCVD* atherosclerotic cardiovascular disease, *DM* diabetes mellitus, *PCSK9-mAb* proprotein convertase subtilisin/kexin type 9 monoclonal antibody

**p < 0.01

### Lp(a) analysis

Lipoprotein (a) concentrations at baseline were available in 73 patients (median Lp(a) 79 nmol/L). Follow-ups were available in 42 patients (median Lp(a) 54.4 nmol/L). In 12 patients, Lp(a) levels were below the detectable range both at baseline and after inclisiran treatment and were therefore excluded from the analysis. The median Lp(a) change from baseline was − 17.3% (95% CI, − 24.6; − 6.4), ranging from a 74.4% reduction to a 29.6% increase in Lp(a) levels from baseline (Supplementary Fig. 5). There was no association between LDL-C change from baseline (%) and baseline Lp(a) levels (Supplementary Fig. 6).

### Safety analysis

Forty-two percent of the patients included in this analysis were on inclisiran monotherapy due to side effects of statins, ezetimibe, bempedoic acid or PCSK9-mAb. As many as 70% of the patients included were statin-intolerant. Against this background, inclisiran was extremely well-tolerated. Only 5.9% of the entire cohort reported side-effects after inclisiran administration. Four patients reported myalgia, four patients experienced injection site reactions and one patient had injection site reactions and dizziness.

## Discussion

In this real-world setting outside controlled clinical trials of patients treated with inclisiran in Germany, we observed a substantial interindividual variability of LDL-C reductions after the first and second administration of the siRNA inclisiran. This finding is consistent with observations reported for other lipid-lowering agents, such as statins, ezetimibe, PCSK9-mAb and, most recently, bempedoic acid [7, 9, 10, 12–15]. Individual patient data analysis of VOYAGER evaluated LDL-C reductions in more than 32,000 statin-treated patients and demonstrated that 5.3–53.3% of these patients were poor-responders [7]. Waterfall plots from the HEYMANS registry—a real-world analysis of the PCSK9-mAb evolocumab—also demonstrated a substantial interindividual variability in LDL-C reductions [16]. Apart from biochemical and molecular properties, there are also other possible factors to explain this observation. In controlled clinical trials, patients exhibit greater adherence to prescribed medications compared to observational studies, as a result of closer supervision, regular follow-up, and higher pre-existing adherence levels [17, 18]. Moreover, patients admitted to special lipid clinics are characterized by multiple drug intolerances. Therefore, this cohort differs from the general population usually treated with LLT.

The median LDL-C reduction of patients who did not receive PCSK9-mAb treatment prior to inclisiran administration was − 41.1% after 3 months and − 28.4% after 9 months. Less effective LDL-C lowering in this cohort could be due to discontinued or reduced dosing of background LLT. Another important finding of this analysis was that PCSK9-mAb pre-treatment was associated with less effective LDL-C reductions (Tables 3, 4). This could be due to patient selection. Patients on PCSK9-mAb are characterized by higher baseline LDL-C levels and less effective LDL-C lowering on other LLT [19, 20]. Moreover, other reasons to switch from PCSK9-mAb to inclisiran were a poor response to PCSK9-mAb treatment and, in some patients, poor tolerability of PCSK9-mAb. Therefore, it cannot be excluded that in this selected patient population, inclisiran is also less effective.

The recently published ORION-3 open-label extension trial does not verify the findings of this study. However, more than two-thirds of the patients in the ORION-3 trial were on concomitant statin therapy, whereas our cohort consisted mainly of statin-intolerant patients.

It is known that PCSK9 inhibition by monoclonal antibodies increases PCSK9 plasma concentrations within the first 3 months after PCSK9-mAb injection due to delayed PCSK9 plasma clearance induced by the PCSK9-antibody complex [21]. This could potentially be a reason as to why PCSK9-mAb pre-treatment was associated with less pronounced LDL-C reduction. To which extent this may influence the magnitude of LDL-C reductions in response to inclisiran and what additional pathways might contribute to the relationship between PCSK9 protein and LDL-C reductions is not fully understood.

Another point worth mentioning is that although PCSK9 is highly specific to the liver, this is not the only tissue where PCSK9 mRNA is expressed. Other tissues and cells, such as the central nervous system, vascular smooth muscle cells (VSMCs), macrophages, endothelial cells, lungs, esophagus, stomach, duodenum, small intestine, colon, rectum, kidneys and pancreas also express PCSK9. In VSMCs, macrophages and endothelial cells, PCSK9 controls the LDL-R expression level similar to hepatic PCSK9 [22, 23]. This may lead to impaired LDL-C clearance, which cannot be remedied through PCSK9 hepatic inhibition alone and could be one explanation why PCSK9-antibodies showed higher efficacy than siRNA inclisiran in the ORION-3 extension trial (although there has been no direct head-to-head comparison between two treatments) [24].

The cohort of this study is highly heterogeneous in terms of concomitant LLT. Patients receiving statin treatment had significantly greater LDL-C reductions than patients not on statins (Table 3, Supplementary Figure S3). This finding is in accordance with a previous publication assessing inclisiran in a real-world cohort [25]. It is well-known that statins induce the expression of the sterol-binding regulatory protein-2 (SREBP-2), a process leading to increased transcription of both LDL-R and PCSK9 mRNA and hence, to elevation of PCSK9 concentration in plasma. Previous studies have also shown that greater LDL-C reductions in response to statins are positively associated with PCSK9 plasma levels [26, 27]. Moreover, it is hypothesized that the relationship between statin therapy and PCSK9 plasma concentrations could be an explanation for variations in LDL-C response to statin treatment [27].

It has been suggested that poor adherence to statins, PCSK9/LDL-R mutations and high Lp(a) levels may lead to a suboptimal response to PCSK9 inhibition [28]. Although the first two factors cannot be ruled out, our observations did not indicate a significant association between Lp(a) levels and the reduction of LDL-C from baseline (Supplementary Figure S6). Further, our study confirmed previous data from ORION-1 on a substantial individual variation in Lp(a) reductions (Supplementary Figure S5) [29]. Further research is necessary to address the discrepancy in LDL-C reductions observed in patients previously treated with PCSK9-mAbs vs. PCSK9-mAb naïve patients. Prospective studies that incorporate PCSK9 measurements may provide significant value in understanding the underlying mechanisms and factors that influence LDL-C response. Additionally, a more in-depth characterization of the patient cohort, including genetic testing, is of paramount importance in identifying genetically determined reasons for high interindividual variations in LDL-C reductions.

Finally, side-effects of inclisiran treatment were rare. Given the fact that around 50% of this cohort are patients with drug intolerances to multiple other lipid-lowering agents, a 6% rate of side effects to inclisiran our study is consistent with a very good tolerability.

## Limitations

This study has several limitations, most of them characteristic for registry studies. First, due to the retrospective design of the study, we cannot control for residual confounding or draw causal conclusions. Second, the study is based on patient-reported information, and we did not measure drug (or metabolite) concentrations. Therefore, we cannot exclude that in some patients, an increase in LDL-C concentrations, especially after the second inclisiran injection, could be due to non-adherence to concomitant LLT. However, waterfall plots show similar variations in patients with background LLT and inclisiran monotherapy (Supplementary Figure S5). Further, the quality of data collected in retrospective registry studies can vary and is generally lower compared to randomized controlled trials or prospective registries.

Apart from methodological limitations, the study’s limited generalizability should also be emphasized as a drawback. The cohort was highly heterogenous and included patients on various background lipid-lowering therapies as well as patients who received inclisiran monotherapy due to side effects of multiple lipid-lowering agents. Moreover, this study reports results from patients admitted to highly specialized lipid clinics. Therefore, a selection bias cannot be excluded. We also did not compare LDL-C reduction in response to siRNA inclisiran vs. other LLT in the same setting. Further, in patients who were pre-treated with PCSK9 antibody, LDL-C levels within the first months of inclisiran injection may be of limited value due to delayed PCSK9 clearance.

Despite the study's limitations, our data provide valuable insights into the performance of inclisiran in a real-world clinical setting. Registry-based studies enable the gathering of information from actual clinical practice, providing a realistic representation of drug performance in real-world scenarios.

## Conclusions

This retrospective, multicenter cohort study reports the first real-world data of LDL-C and Lp(a) lowering after administration of the siRNA inclisiran outside of controlled clinical trials in Germany. The high interindividual variability of LDL-C responses demonstrates the need to “treat-to-target” and supports the concept of “individualized lipid-lowering therapy”.

### Supplementary Information

Below is the link to the electronic supplementary material.

Supplementary file1 (PDF 504 KB)

## References

[1] Mach F, Baigent C, Catapano AL (2020). 2019 ESC/EAS Guidelines for the management of dyslipidaemias: lipid modification to reduce cardiovascular risk. Eur Heart J.

[2] Dyrbuś K, Gąsior M, Penson P, Ray KK, Banach M (2020). Inclisiran—new hope in the management of lipid disorders?. J Clin Lipidol.

[3] Kosmas CE, Pantou D, Sourlas A, Papakonstantinou EJ, Echavarria Uceta R, Guzman E (2021). New and emerging lipid-modifying drugs to lower LDL cholesterol. Drugs Context.

[4] Brandts J, Ray KK (2020). Small interfering RNA to proprotein convertase subtilisin/kexin type 9: transforming LDL-cholesterol-lowering strategies. Curr Opin Lipidol.

[5] Seidah NG, Awan Z, Chrétien M, Mbikay M (2014). PCSK9: a key modulator of cardiovascular health. Circ Res.

[6] Lamb YN (2021). Inclisiran: first approval. Drugs.

[7] Karlson BW, Wiklund O, Palmer MK, Nicholls SJ, Lundman P, Barter PJ (2016). Variability of low-density lipoprotein cholesterol response with different doses of atorvastatin, rosuvastatin, and simvastatin: results from VOYAGER. Eur Heart J Cardiovasc Pharmacother.

[8] Qamar A, Giugliano RP, Keech AC (2019). Interindividual variation in low-density lipoprotein cholesterol level reduction with evolocumab: an analysis of FOURIER trial data. JAMA Cardiol.

[9] Warden BA, Cardiology BA, Purnell JQ, Duell PB, Fazio S (2022). Real-world utilization of bempedoic acid in an academic preventive cardiology practice. J Clin Lipidol.

[10] Descamps O, Tomassini JE, Lin J (2015). Variability of the LDL-C lowering response to ezetimibe and ezetimibe + statin therapy in hypercholesterolemic patients. Atherosclerosis.

[11] Gemeinsamer Bundesausschuss. Arzneimittel-Richtlinie/Anlage III: Nummer 35c—Inclisiran. https://www.g-ba.de/beschluesse/5072/. Accessed 3 July 2023

[12] Ridker PM, Mora S, Rose L, JUPITER Trial Study Group (2016). Percent reduction in LDL cholesterol following high-intensity statin therapy: potential implications for guidelines and for the prescription of emerging lipid-lowering agents. Eur Heart J.

[13] Boekholdt SM, Hovingh GK, Mora S (2014). Very low levels of atherogenic lipoproteins and the risk for cardiovascular events. J Am Coll Cardiol.

[14] Lütjohann D, Stellaard F, Mulder MT, Sijbrands EJG, Weingärtner O (2019). The emerging concept of “individualized cholesterol-lowering therapy”: a change in paradigm. Pharmacol Ther.

[15] Koren MJ, Lundqvist P, Bolognese M (2014). Anti-PCSK9 monotherapy for hypercholesterolemia. J Am Coll Cardiol.

[16] Ray KK, Bruckert E, Peronne-Filardi P (2023). Long-term persistence with evolocumab treatment and sustained reductions in LDL-cholesterol levels over 30 months: final results from the European observational HEYMANS study. Atherosclerosis.

[17] Atar D, Ong S, Lansberg PJ (2015). Expanding the evidence base: comparing randomized controlled trials and observational studies of statins. Am J Ther.

[18] van Onzenoort HAW, Menger FE, Neef C (2011). Participation in a clinical trial enhances adherence and persistence to treatment: a retrospective cohort study. Hypertension.

[19] Zafrir B, Jubran A (2018). Lipid-lowering therapy with PCSK9-inhibitors in the real-world setting: two-year experience of a regional lipid clinic. Cardiovasc Ther.

[20] Altschmiedová T, Todorovová V, Šnejdrlová M, Šatný M, Češka R (2022). PCSK9 inhibitors in real-world practice: analysis of data from 314 patients and 2 years of experience in a center of preventive cardiology. Curr Atheroscler Rep.

[21] Oleaga C, Shapiro MD, Hay J (2021). Hepatic sensing loop regulates PCSK9 secretion in response to inhibitory antibodies. J Am Coll Cardiol.

[22] Xia XD, Peng ZS, Gu HM, Wang M, Wang GQ, Zhang DW (2021). Regulation of PCSK9 expression and function: mechanisms and therapeutic implications. Front Cardiovasc Med.

[23] Schlüter KD, Wolf A, Schreckenberg R (2020). Coming back to physiology: extra hepatic functions of proprotein convertase subtilisin/kexin type 9. Front Physiol.

[24] Ray KK, Troquay RPT, Visseren FLJ (2023). Long-term efficacy and safety of inclisiran in patients with high cardiovascular risk and elevated LDL cholesterol (ORION-3): results from the 4-year open-label extension of the ORION-1 trial. Lancet Diabetes Endocrinol.

[25] Padam P, Barton L, Wilson S (2022). Lipid lowering with inclisiran: a real-world single-centre experience. Open Heart.

[26] Sahebkar A, Simental-Mendía LE, Guerrero-Romero F, Golledge J, Watts GF (2015). Effect of statin therapy on plasma proprotein convertase subtilisin kexin 9 (PCSK9) concentrations: a systematic review and meta-analysis of clinical trials. Diabetes Obes Metab.

[27] Taylor BA, Thompson PD (2016). Statins and their effect on PCSK9-impact and clinical relevance. Curr Atheroscler Rep.

[28] Ouyang M, Li C, Hu D, Peng D, Yu B (2023). Mechanisms of unusual response to lipid-lowering therapy: PCSK9 inhibition. Clin Chim Acta.

[29] Ray KK, Stoekenbroek RM, Kallend D (2018). Effect of an siRNA therapeutic targeting PCSK9 on atherogenic lipoproteins: prespecified secondary end points in ORION 1. Circulation.

