# Supplementary material for: High interindividual variability in LDL-cholesterol reductions after inclisiran administration in a real-world multicenter setting in Germany
Source: Clin Res Cardiol. 2023 Jul 9;112(11):1639–49. doi: 10.1007/s00392-023-02247-8 (PMC10584696; doi:10.1007/s00392-023-02247-8)
Supplement: Supplementary file 1 — Supplementary file1 (PDF 504 KB) [file 392_2023_2247_MOESM1_ESM.pdf]

Supplementary Material

High interindividual variability in LDL-cholesterol reductions after inclisiran administration in a “real-world” setting in Germany

Makhmudova U[1,8,9], Schatz U[2], Perakakis N[2,3,4], Kassner U[5], Schumann F[5], Axthelm C[6], Stürzebecher P[7], Sinning DL[8], Doevelaar A[10], Rohn B[10], Westhoff T[10], Vogt A[11], Scholl M[12], Kästner U[12], Geiling J-A[1], Stach K[13], Mensch J[14], Lorenz E[15],Paitazoglou C[16,17], Eitel I[16,17], Bäßler A[18], Steinhagen-Thiessen E[5,9,14], Koenig W[15,19,20], Schulze PC[1], Landmesser U[8,9], Laufs U[7], Weingärtner O[1] for the GERMAN INCLISIRAN NETWORK (GIN)

[1]Klinik für Innere Medizin I, University Hospital Jena, Jena

[2]Department of Internal Medicine III, University Hospital Carl Gustav Carus, Technische Universität Dresden, Dresden, Germany

[3]Paul Langerhans Institute Dresden (PLID), Helmholtz Center Munich, University Hospital and Faculty of Medicine, TU Dresden, Dresden, Germany

[4]German Center for Diabetes Research (DZD e.V.), Neuherberg, Germany

[5]Clinic for Endocrinology and Metabolic Medicine, Charité – University Medicine Berlin, Berlin

[6]Cardiologicum Dresden and Pirna, Dresden, Germany

[7]Klinik und Poliklinik für Kardiologie, Universitätsklinikum Leipzig, Germany;

[8]Deutsches Herzzentrum der Charité, Department of Cardiology, Angiology and Intensive Care Medicine, Berlin, Germany

[9]Friede Springer Cardiovascular Prevention Center, Berlin, Germany

[10]Medical Clinic I, Marien Hospital Herne, University Hospital of the Ruhr-University of Bochum, Herne, Germany

[11]Medical Clinic and Policlinic IV, University Hospital München, Germany

[12]Nephrocare Mühlhausen GmbH, Medical Care Centre, Mühlhausen/Thüringen, Germany

[13]University Hospital Mannheim, Medical Clinic V, Mannheim, Germany

[14]Institute for Clinical Chemistry, University Medicine Rostock, Rostock, Germany

[15]Deutsches Herzzentrum München, Technische Universität München, Munich, Germany

[16]Medizinische Klinik II, Universitätsklinikum Schleswig-Holstein, Lübeck, Germany

[17]German Centre for Cardiovascular Research (DZHK), partner site Hamburg-Kiel-Lübeck, Lübeck, Germany

[18] Klinik für Innere Medizin II, Universitätsklinikum Regensburg, Regensburg, Germany

[19]German Centre for Cardiovascular Research (DZHK), partner site Munich Heart Alliance, Munich, Germany

[20]Institute of Epidemiology and Medical Biometry, University of Ulm, Ulm, Germany

German Inclisiran Network

14 sites

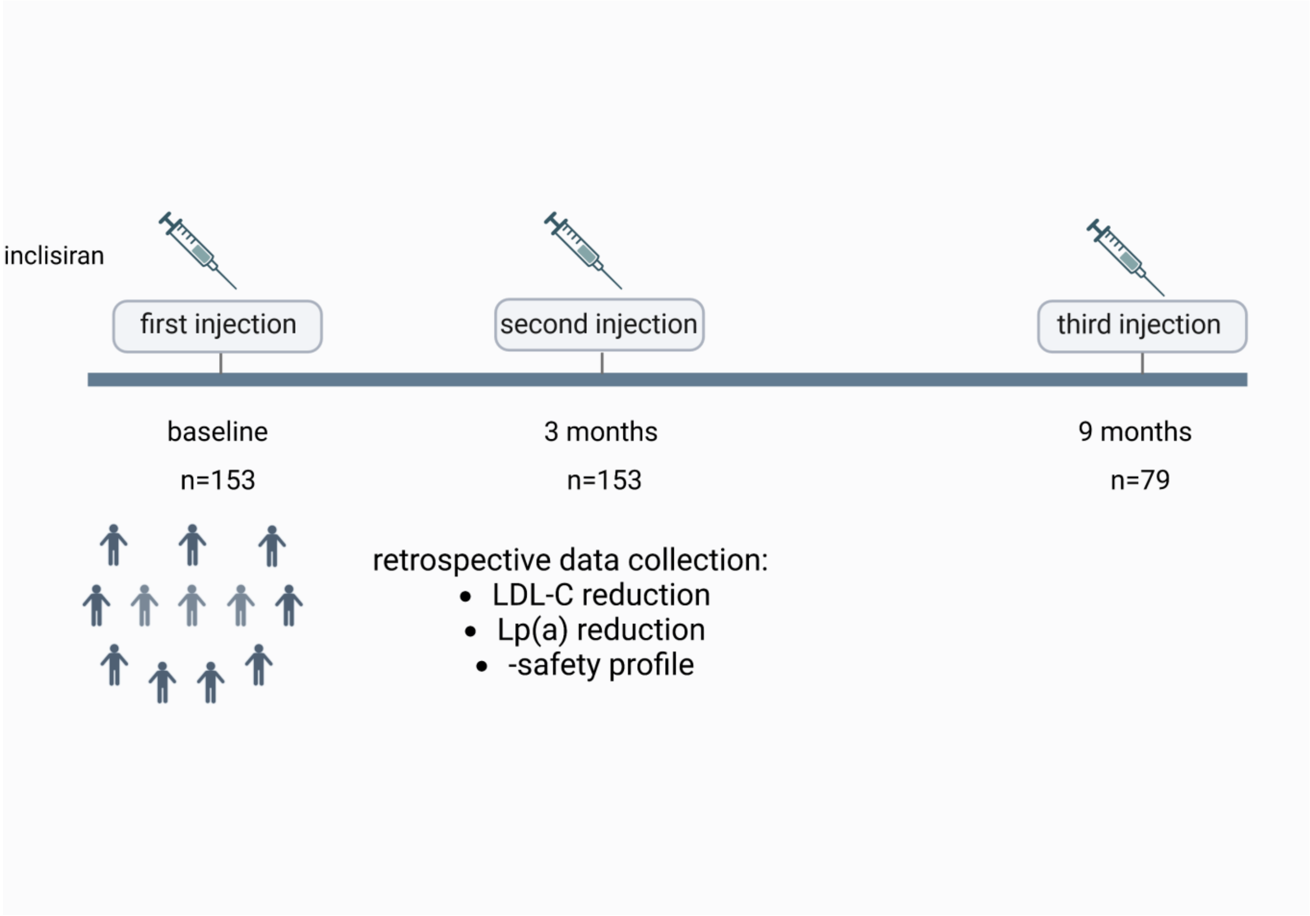

Supplementary figure 1. Study description.Created with BioRender.com

	Center
**1**	University Hospital Leipzig
**2**	University Hospital Carl Gustav Carus, Dresden
**3**	Munich Heart Center
**4**	Charité University Hospital (Deutsches Herzzentrum der Charité)
**5**	Charité University Hospital (Department of Endocrinology)
**6**	Jena University Hospital
**7**	Dresden outpatient clinic “Cardiologicum Dresden und Pirna”
**8**	Nephocare Mühlhausen GmBH
**9**	University Hospital of the Ruhr-University of Bochum
**10**	University Medicine Rostock
**11**	University Hospital Munich
**12**	University Hospital Mannheim
**13**	University Hospital Lübeck
**14**	University Hospital Regensburg

Supplementary Table 1. List of participating sites.

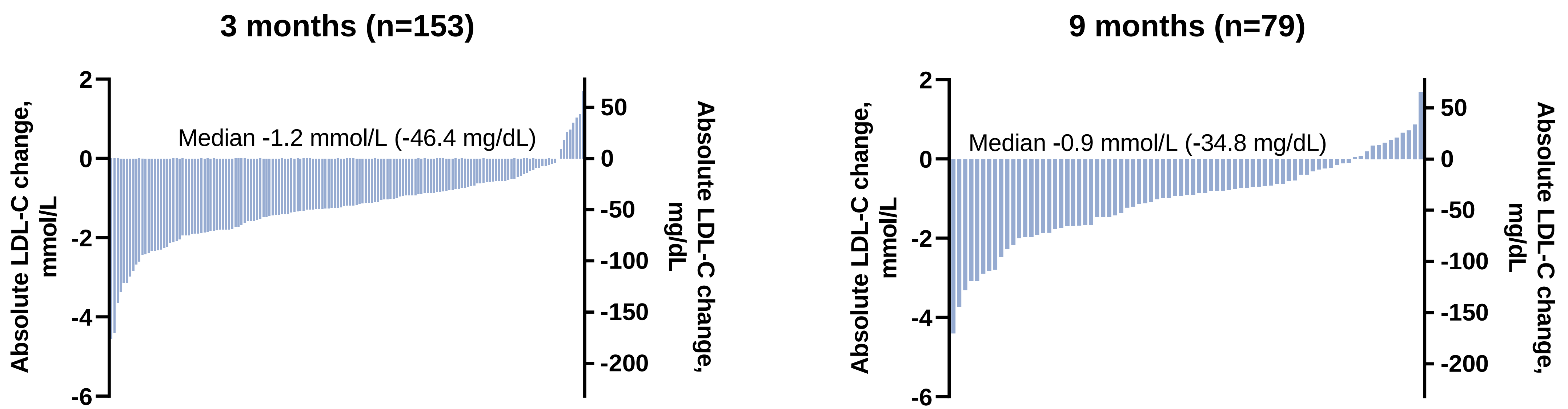

Supplementary figure 2. Absolute changes in LDL-C at 3 and 9 months.

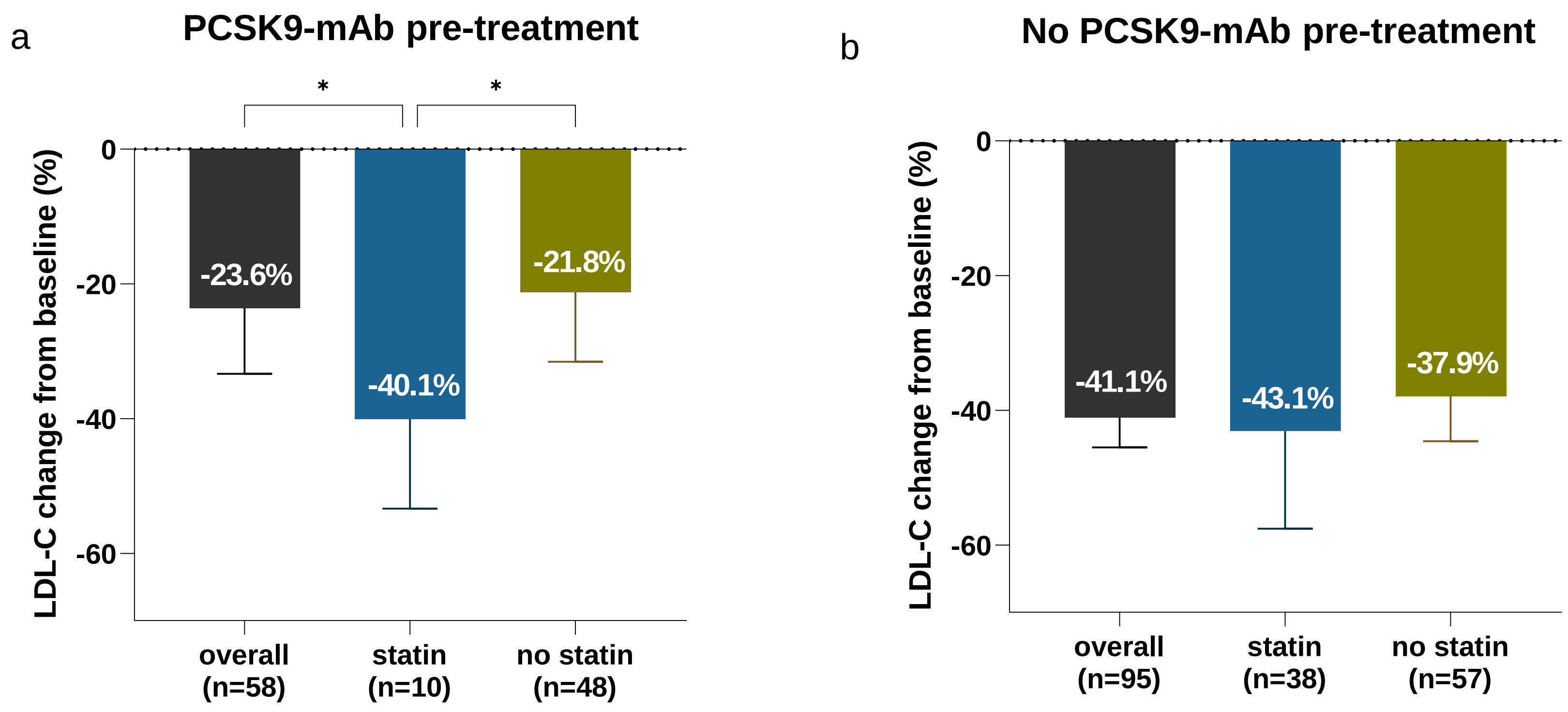

Supplementary figure 3. LDL-C change from baseline (%) in patients PCSK9-mAb history (a) vs no-PCSK9-mAb history (b) shown as median and IQR.

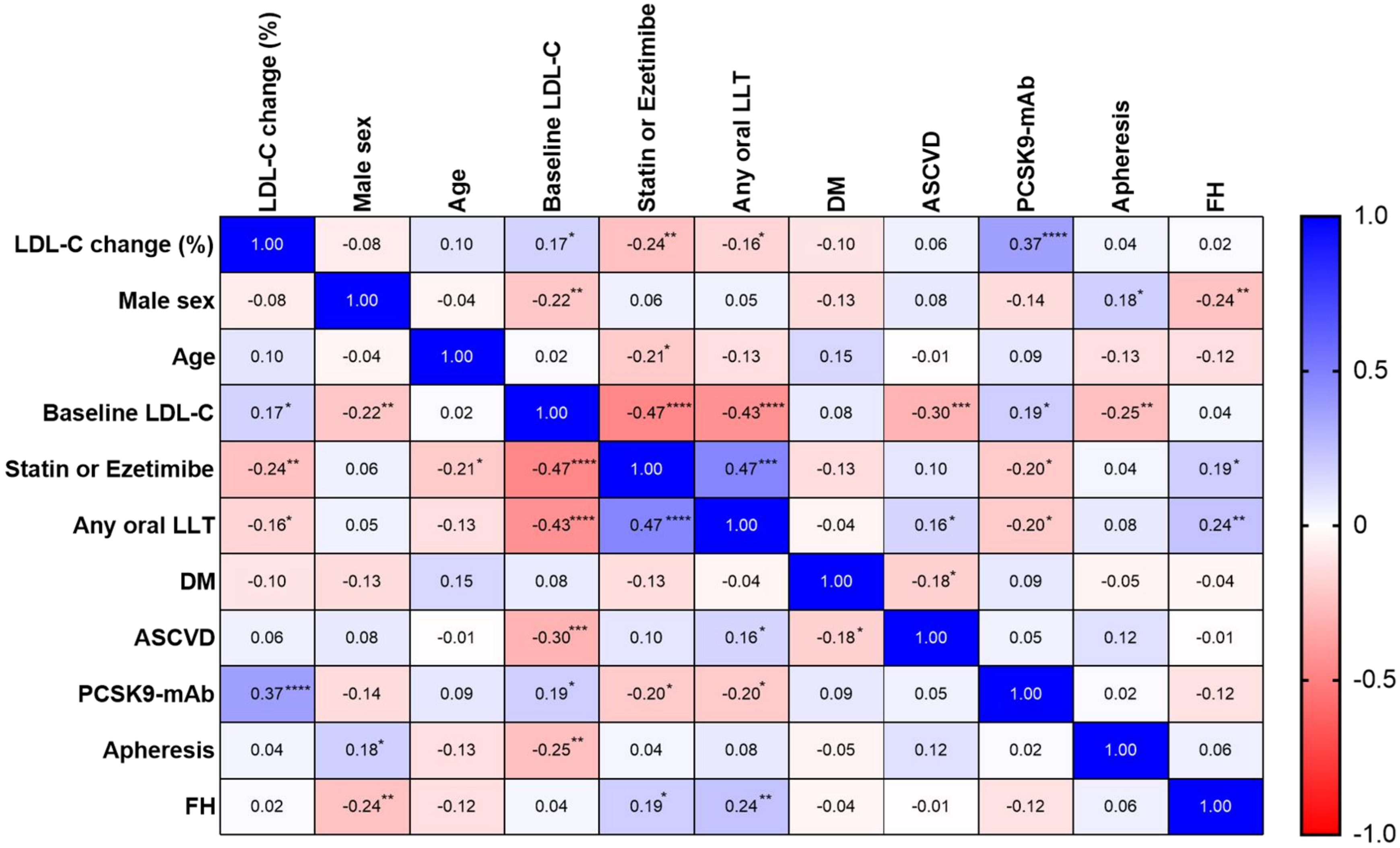

Supplementary figure 4. Heatmap correlations between relative LDL-C change from baseline (%) and demographic and clinical parameters. *p<0.05, **p<0.01, ***p<0.001, ****p<0.0001

ASCVD, atherosclerotic cardiovascular disease; DM, diabetes mellitus, PCSK9-mAb, proprotein convertase subtilisin/kexin type 9 monoclonal antibody.

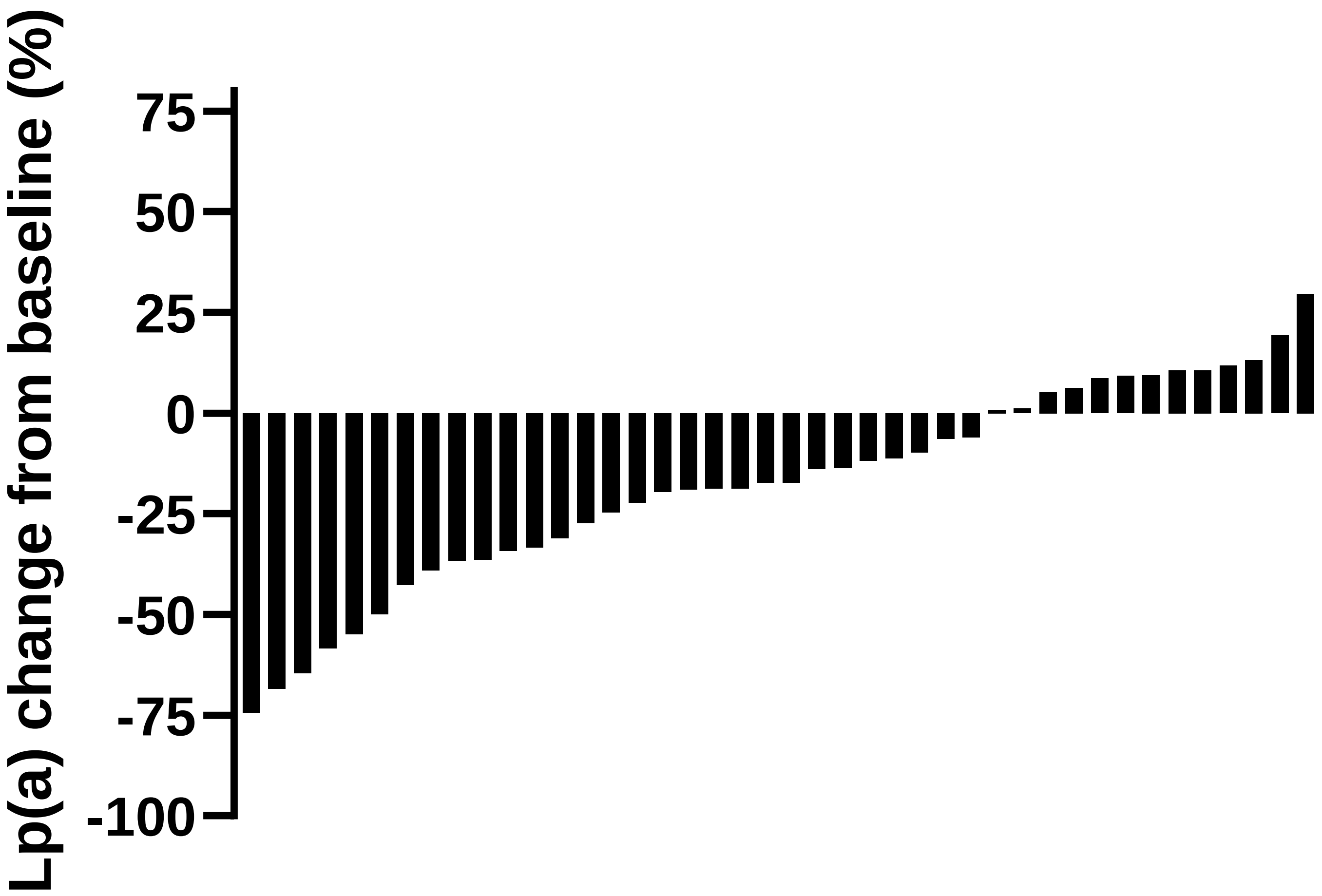

Supplementary figure 5. Waterfall plot of Lp(a) change from baseline (%) after inclisiran injection (3 months). Waterfall plot shows individual variations in Lp(a) reduction in 42 patients, qualified for the analysis.

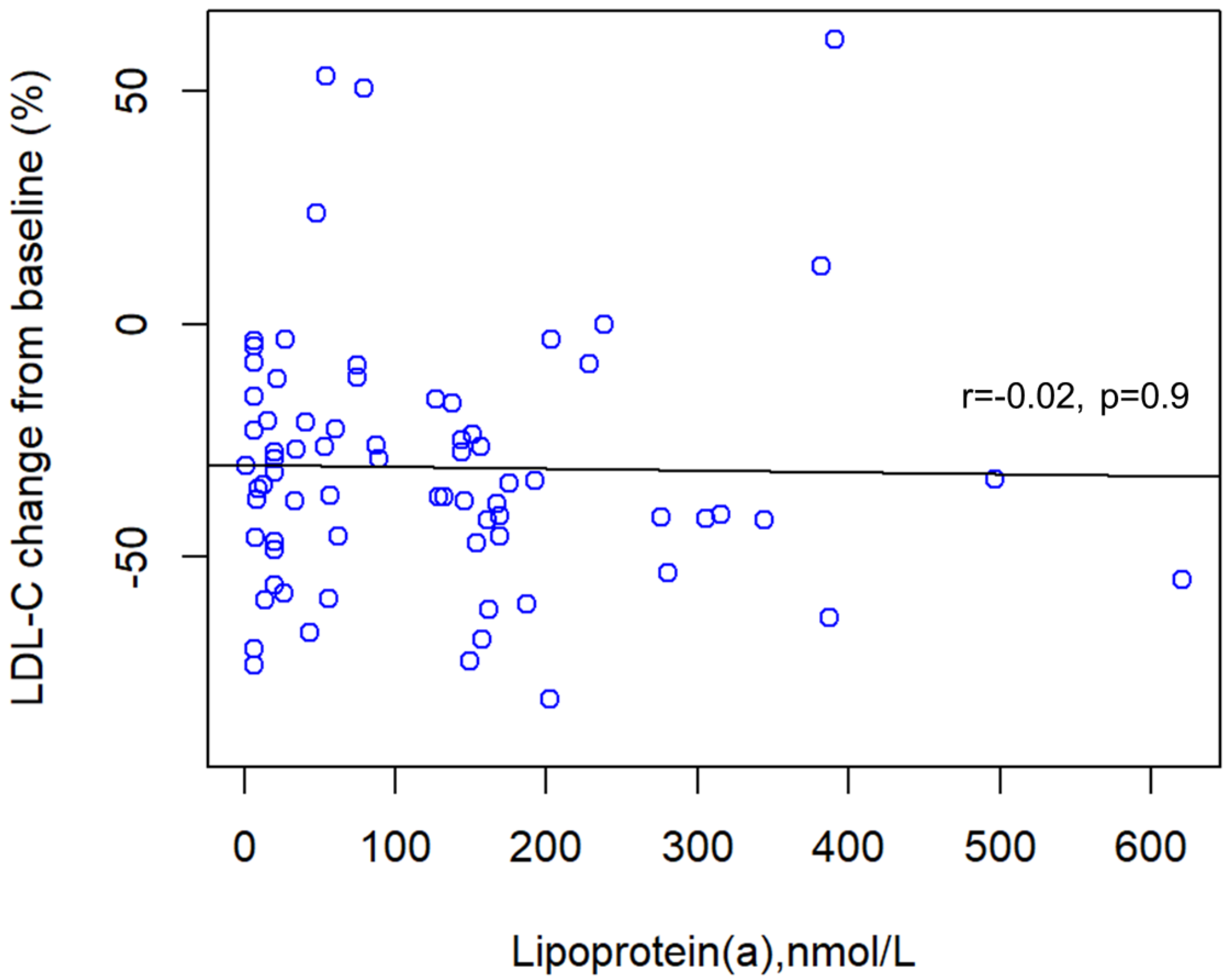

Supplementary figure 6. Correlation between LDL-C change from baseline (%) and baseline lipoprotein(a) concentrations.
